# Crosstalk of Cytokinin with Ethylene and Auxin for Cell Elongation Inhibition and Boron Transport in Arabidopsis Primary Root under Boron Deficiency

**DOI:** 10.3390/plants11182344

**Published:** 2022-09-08

**Authors:** María Begoña Herrera-Rodríguez, Juan José Camacho-Cristóbal, Rafael Barrero-Rodríguez, Jesús Rexach, María Teresa Navarro-Gochicoa, Agustín González-Fontes

**Affiliations:** Departamento de Fisiología, Anatomía y Biología Celular, Universidad Pablo de Olavide, E-41013 Sevilla, Spain

**Keywords:** *ACS11* gene, *AUX1* gene, boron transporters, plant hormones, root elongation

## Abstract

Several studies have shown the role of phytohormones in the regulation of root growth of Arabidopsis plants under boron (B) deficiency. Ethylene and auxin play an important role in the control of Arabidopsis primary root cell elongation under short-term B deprivation, whereas cytokinins regulate root growth inhibition under B deficiency by controlling meristem cell proliferation. In this work, we study the possible interaction among cytokinin, ethylene, and auxin in the primary root response to B-deprivation treatment, as well as their possible role in B uptake and transport. Wild type (WT) and two mutants related to auxin and ethylene (*aux1* and *acs11*) Arabidopsis plants were grown in control (10 µM B) or B starvation (0 µM B) treatment, in the absence or presence of *trans*-zeatin, and their primary root growth was analyzed. The possible interaction between these hormones was also studied by analyzing *AUX1* gene expression in the *acs11* mutant and *ACS11* gene expression in the *aux1* mutant. The GUS reporter lines ARR5::GUS, IAA2::GUS, and EBS::GUS were used to observe changes in cytokinin, auxin, and ethylene levels in the root, respectively. The results of this work suggest that cytokinin inhibits root cell elongation under B deficiency through two different mechanisms: (i) an ethylene-dependent mechanism through increased expression of the *ACS11* gene, which would lead to increased ethylene in the root, and (ii) an ethylene-independent mechanism through decreased expression of the *AUX1* gene, which alters auxin signaling in the meristematic and elongation zones and stele. We also report that changes in the expression of several B transporters occur in response to auxin, ethylene, and cytokinin that may affect the plant B content.

## 1. Introduction

Boron (B) is an essential element for plant development, and its deficiency limits crop productivity worldwide, producing significant economic losses [1,2,3,4,5]. For instance, B deficiency has been reported to reduce the yield of cotton (*Gossypium hirsutum*), rice (*Oryza sativa*), maize (*Zea mays*), wheat (*Triticum aestivum*), and soybean (*Glycine max*) crops [6]. Low B availability affects several physiological and metabolic processes in plants, such as cell wall and plasma membrane structure and function, phenolic and nitrogen metabolisms, secondary metabolism and oxidative stress, gene expression, and shoot and root growth [7,8,9,10,11,12], among others.

Depending on B availability in the soil, its uptake as boric acid by root cells and xylem loading can take place by three different mechanisms: (i) passive diffusion through the plasma membrane, (ii) facilitated diffusion carried out by nodulin intrinsic protein (NIP) channels, and (iii) energy-dependent high-affinity transport, mediated via BOR transporters and induced under low B availability [8,9,13,14,15].

The best-known function of B in vascular plants is its structural role in the cell wall through the stabilization of molecules containing *cis*-diol groups (borate esters with apiose residues of two rhamnogalacturonan II (RG-II) monomers) [16,17,18]. Consequently, plants subjected to B starvation normally show altered cell wall phenotypes and growth defects [19,20]. For instance, the most rapid response to B deficiency in vascular plants is the inhibition of root elongation in both the main and lateral roots [21].

Plants adapt their growth to changes in nutrient availability, and hormones act as endogenous regulatory factors that control this response [22]. Among others, there are several studies showing that cytokinins ─alone or together with other hormones such as ethylene, auxin, or ABA─ play a particular role in controlling plant responses to different nutritional stresses [23,24,25,26,27,28,29,30]. Total root growth depends on two processes: cell division (mostly in the root meristematic region) and enlargement (in the elongation zone). In Arabidopsis plants, several physiological studies support that B deficiency causes a decrease in primary root cell elongation in a process controlled by the interaction between ethylene (via the ACC synthase (ACS) isoform ACS11) and auxin (through the auxin influx-carrier AUX1), among other possible intermediates [31,32,33]. This leads to a lower growth of the primary root. Moreover, a cytokinin-mediated inhibition of cell proliferation in the root meristematic region contributing to root growth inhibition in Arabidopsis was reported in plants subjected to B deprivation [34,35]. It is well known that cytokinin negatively regulates root growth by interacting with auxin ─through the auxin influx-carrier AUX1─, and ethylene [36,37,38,39,40].

Therefore, considering all this evidence, the aim of this work has been to investigate the possible interaction between cytokinin, ethylene, and auxin in Arabidopsis primary root response to B-deprivation treatment, as well as their possible role in B uptake and transport. Our results show that cytokinin inhibits root cell elongation under B deficiency through two different mechanisms: (i) an ethylene-dependent mechanism by increasing *ACS11* gene expression, which would lead to increased ethylene in the root, and (ii) an ethylene-independent mechanism by decreasing *AUX1* gene expression, which alters auxin signaling in the meristematic and elongation zones and stele. In addition, changes in the expression of several B transporters occur in response to auxin, ethylene, and cytokinin that may affect the plant B content. Further studies on the regulation of B transporters by these hormones would be interesting to continue this line of work.

## 2. Results

In a first experimental approach, a temporal analysis of primary root elongation was carried out in Arabidopsis seedlings transferred to control (10 μM B) or B-deficient (0 μM B) medium for 96 h in the absence or presence of different concentrations of *trans*-zeatin (Figure S1). As expected, B deficiency caused a significant inhibition of primary root elongation when compared with the control treatment from 24 h onwards (Figure S1). Interestingly, the exogenous application of 50 nM *trans*-zeatin caused a slight decrease in primary root elongation under control conditions from 24 h onwards (Figure S1), whereas it led to a slight increase in primary root elongation under B deficiency from 48 h onwards (Figure S1). Taking into account these results, for the following experiments 48 h of B treatments with or without 50 nM *trans*-zeatin were chosen.

### 2.1. Effect of Cytokinin Treatment on Primary Root Growth of Auxin and Ethylene Arabidopsis Mutants under B Deficiency

**Figure 1:**
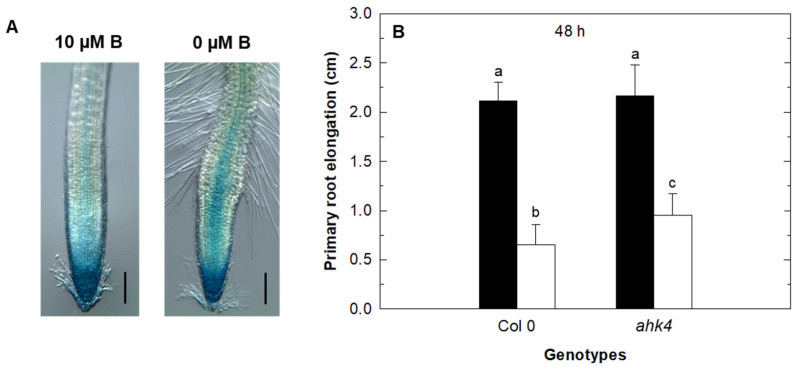
Changes in cytokinin signaling affect primary root elongation under B deficiency. (**A**) GUS expression in the roots of ARR5::GUS seedlings grown in control (10 μM B) or B-deficient (0 μM B) medium for 48 h. Images are representative individuals from two independent experiments with at least 11 seedlings examined for each experiment. Scale bar = 100 μm. (**B**) Primary root elongation in Arabidopsis wild type (Col 0) and ahk4 mutant measured in roots grown in control (black bars) or B-deficient (white bars) treatment for 48 h. Results are given as means ± SD (*n* = 24 separate plants). Different letters indicate statistically significant differences between treatments and genotypes according to ANOVA with Tukey’s HSD test (*p* < 0.01).

It has recently been shown that cytokinin regulates root growth inhibition under B deficiency by controlling meristem activity [35]. These authors, using the Arabidopsis reporter line ARR5::GUS to report changes in cytokinin signaling in seedlings subjected to B deficiency, observed increased GUS signaling localized in the root vascular cylinder (or stele) and root meristematic zone when seedlings were grown under B deficiency [35]. Similar results were obtained under our growing conditions (Figure 1A). This fact, together with a lower inhibition in primary root elongation observed in the ahk4 mutant (affected in cytokinin perception) under B starvation treatment could indicate that this hormone would be controlling primary root elongation under these conditions (Figure 1B).

To further explore the possible mediation of cytokinin in the effect of B deficiency on primary root elongation and its interaction with ethylene and auxin, phytohormones that regulate this process [31,32,33], Arabidopsis mutants defective in auxin (*aux1*) or ethylene (*acs11*) response were grown in control or B starvation treatment in the absence or presence of *trans*-zeatin for 2 days. In both mutants, less primary root growth was observed when compared with their respective wild types (WT) in all the treatments analyzed (Figures S2 and S3).

**Figure 2:**
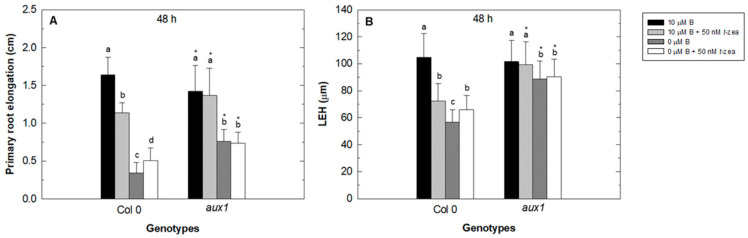
Effect of cytokinin treatment on primary root growth of Arabidopsis wild type (Col 0) and *aux1* mutant under B deficiency. Primary root elongation in Arabidopsis wild type (Col 0) and *aux1* mutant (**A**) and the LEH parameter (**B**) were measured in roots grown in control (black bars), control plus 50 nM *trans*-zeatin (light grey bars), B-deficient (dark grey bars), and B-deficient plus 50 nM *trans*-zeatin (white bars) treatment after 48 h. Results are given as means ± SD (*n* = 24 separate plants). Different letters indicate statistically significant differences between treatments for each genotype according to ANOVA with Tukey’s HSD test (*p* < 0.01). Asterisks indicate statistically significant differences between genotypes for each treatment according to Student’s *t*-test (*p* < 0.05).

Primary root elongation in Col 0 (WT) was more inhibited by B starvation treatment than the *aux1* mutant after 48 h of treatment (Figure 2A). As expected, external addition of *trans*-zeatin to B-sufficient WT plants resulted in a clear reduction of primary root elongation. However, this addition to B-deficient WT plants increased the primary root growth after 48 h of treatment (Figure 2A). No changes in primary root elongation were observed in the *aux1* mutant caused by the external addition of *trans*-zeatin (Figure 2A). In this study, the length of the first epidermal cell with a visible root hair bulge (LEH; [41,42]) was measured after 48 h of treatments in order to evaluate its effects on root cell elongation [43], and similar results were obtained to those for primary root elongation (Figure 2B).

**Figure 3:**
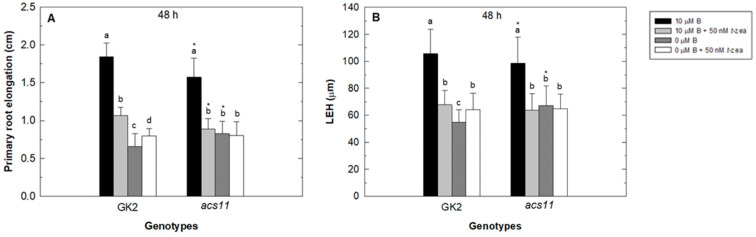
Effect of cytokinin treatment on primary root growth of Arabidopsis wild type (GK2) and *acs11* mutant under B deficiency. Primary root elongation in Arabidopsis wild type (GK2) and *acs11* mutant (**A**) and LEH parameter (**B**) were measured in roots grown in control (black bars), control plus 50 nM *trans*-zeatin (light grey bars), B-deficient (dark grey bars), and B-deficient plus 50 nM *trans*-zeatin (white bars) treatment after 48 h. Results are given as means ± SD (*n* = 24 separate plants). Different letters indicate statistically significant differences between treatments for each genotype according to ANOVA with Tukey’s HSD test (*p* < 0.01). Asterisks indicate statistically significant differences between genotypes for each treatment according to Student’s *t*-test (*p* < 0.05).

As occurred in the *aux1* mutant, primary root growth in the *acs11* mutant was less affected than in GK2 (WT) under B deficiency after 48 h of treatment (Figure 3A). External addition of *trans*-zeatin to B-sufficient plants reduced primary root elongation in both genotypes, while when added to B-deficient plants it caused an increase in primary root elongation only in GK2 (Figure 3A). Again, similar results to those for primary root elongation were obtained in the LEH measurements in all treatments analyzed (Figure 3B).

### 2.2. Effect of Cytokinin Treatment on Total B Content and Gene Expression of B Transporters of Auxin and Ethylene Arabidopsis Mutants under B Deficiency

**Figure 4:**
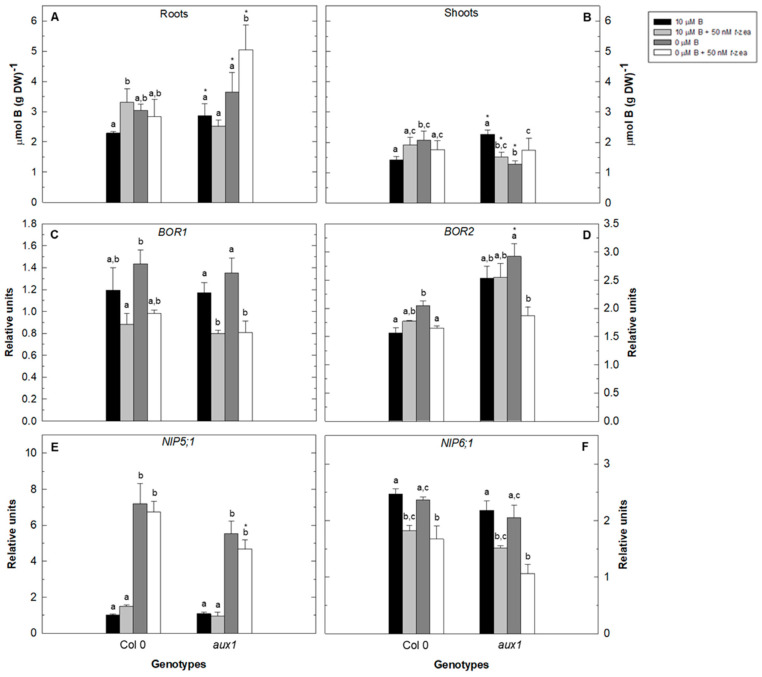
Effect of cytokinin treatment on total B content and gene expression of B transporters of Arabidopsis wild type (Col 0) and *aux1* mutant under B deficiency. Total B content was measured in roots (**A**) and shoots (**B**) of Arabidopsis wild type (Col 0) and *aux1* mutant grown in control (black bars), control plus 50 nM *trans*-zeatin (light grey bars), B-deficient (dark grey bars), and B-deficient plus 50 nM *trans*-zeatin (white bars) treatment after 48 h. Quantitative RT-PCR analysis of *BOR1* (**C**), *BOR2* (**D**), *NIP5;1* (**E**), and *NIP6;1* (**F**) transcript levels in roots were measured in the same treatments. Results are given as means ± SD (*n* = 3 separate pools). Different letters indicate statistically significant differences between treatments for each genotype according to ANOVA with Tukey’s HSD test (*p* < 0.05). Asterisks indicate statistically significant differences between genotypes for each treatment according to Student’s *t*-test (*p* < 0.05).

**Figure 5:**
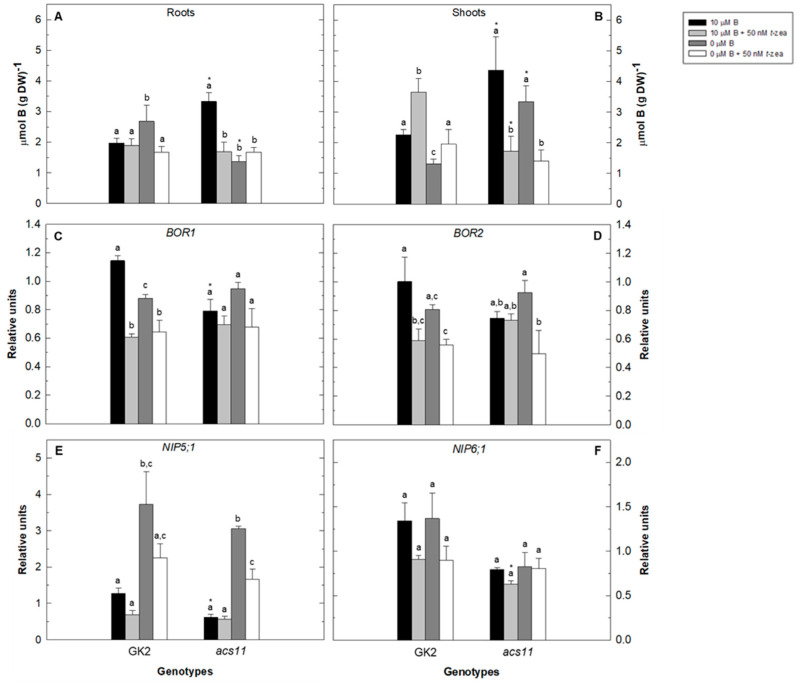
Effect of cytokinin treatment on total B content and gene expression of B transporters of Arabidopsis wild type (GK2) and *acs11* mutant under B deficiency. Total B content was measured in roots (**A**) and shoots (**B**) in Arabidopsis wild type (GK2) and *acs11* mutant grown in control (black bars), control plus 50 nM *trans*-zeatin (light grey bars), B-deficient (dark grey bars), and B-deficient plus 50 nM *trans*-zeatin (white bars) treatment after 48 h. Quantitative RT-PCR analysis of *BOR1* (**C**), *BOR2* (**D**), *NIP5;1* (**E**), and *NIP6;1* (**F**) transcript levels in roots were measured in the same treatments. Results are given as means ± SD (*n* = 3 separate pools). Different letters indicate statistically significant differences between treatments for each genotype according to ANOVA with Tukey’s HSD test (*p* < 0.05). Asterisks indicate statistically significant differences between genotypes for each treatment according to Student’s *t*-test (*p* < 0.05).

Because the external addition of cytokinin alters primary root growth in WT plants and in the *acs11* mutant, we proceeded to analyze the total B content in plants grown in the control or B-deficient treatment in the absence or presence of *trans*-zeatin for 48 h (Figure 4 and Figure 5).

Total root B content was higher in the *aux1* mutant than in Col 0, except for the control treatment in the presence of *trans*-zeatin, while in shoots this fact only occurred in the control treatment (Figure 4A,B). Nevertheless, these results did not correlate with the gene expression of B transporters in roots because, except for *BOR2* expression, the other genes analyzed showed similarities between both genotypes (Figure 4C–F). Addition of *trans*-zeatin to both B-sufficient WT plants and B-deficient *aux1* mutants resulted in a clear increase of total root B content (Figure 4A). This increase was less evident in the shoots of both genotypes (Figure 4B). The strong induction in *NIP5;1* gene expression upon B-starvation treatment in the absence or presence of *trans*-zeatin did not translate into a significant increase in total root B content, except for the *aux1* mutant treated with B deficiency and the cytokinin (Figure 4A,E). Furthermore, the presence of *trans*-zeatin in the control and B-deficient treatments repressed the expression of *BOR1* and *NIP6;1* genes in both genotypes (Figure 4C,F).

When we analyzed the results in the *acs11* mutant, a completely different response than WT was observed (Figure 5). Thus, in roots, the B-deficient treatment as well as the addition of *trans*-zeatin to the control treatment caused a decrease in total B content in the mutant, and no significant changes were observed when *trans*-zeatin was added to the B-starvation treatment (Figure 5A). In shoots, the addition of *trans*-zeatin to both the control and B-deficient treatments caused a drastic reduction in total B content in the *acs11* mutant and an increase in WT (Figure 5B). Gene expression of B transporters was also analyzed (Figure 5C–F). It is noteworthy that all the transporters showed lower expression in the *acs11* mutant in the control treatment, being significant in *BOR1* and *NIP5;1* (Figure 5C,E). As expected, *NIP5;1* gene expression was strongly induced by B deficiency in both genotypes, being more evident in the *acs11* mutant (Figure 5E). This large induction in *NIP5;1* gene expression did not lead to a higher total root B content in the mutant (Figure 5A). In WT plants, the addition of *trans*-zeatin to the control treatment repressed *BOR1* and *BOR2* gene expression, whereas adding it to the B-starvation treatment reduced significantly only *BOR1* gene expression (Figure 5C,D). In the *acs11* mutant, addition of the hormone to the B-deficient treatment decreased *BOR2* and *NIP5;1* gene expression (Figure 5D,E). No significant changes in *NIP6;1* transcript level were observed in all treatments in both genotypes (Figure 5F).

### 2.3. Interaction among Cytokinin, Auxin, and Ethylene under B Deficiency

**Figure 6:**
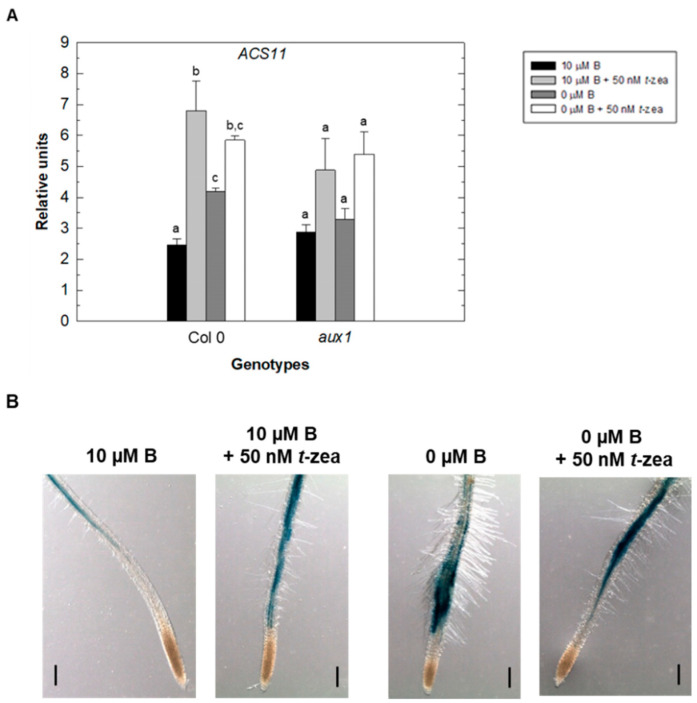
Effect of cytokinin treatment on the expression of the *ACS11* gene in Arabidopsis wild type (Col 0) and *aux1* roots. (**A**) Quantit (dative RT-PCR analysis of *ACS11* transcript levels in control (black bars), control plus 50 nM *trans*-zeatin (light grey bars), B-deficient (dark grey bars), and B-deficient plus 50 nM *trans*-zeatin (white bars) treatment after 48 h. Results are given as means ± SD (*n* = 3 separate pools). Different letters indicate statistically significant differences between treatments for each genotype according to ANOVA with Tukey’s HSD test (*p* < 0.05). (**B**) GUS expression in the roots of ACS11::GUS seedlings grown in control (10 μM B) or B-deficient (0 μM B) medium in the presence or absence of *trans*-zeatin 50 nM for 48 h. Images are representative individuals from two independent experiments with at least 15 seedlings examined for each experiment. Scale bar = 200 μm.

**Figure 7:**
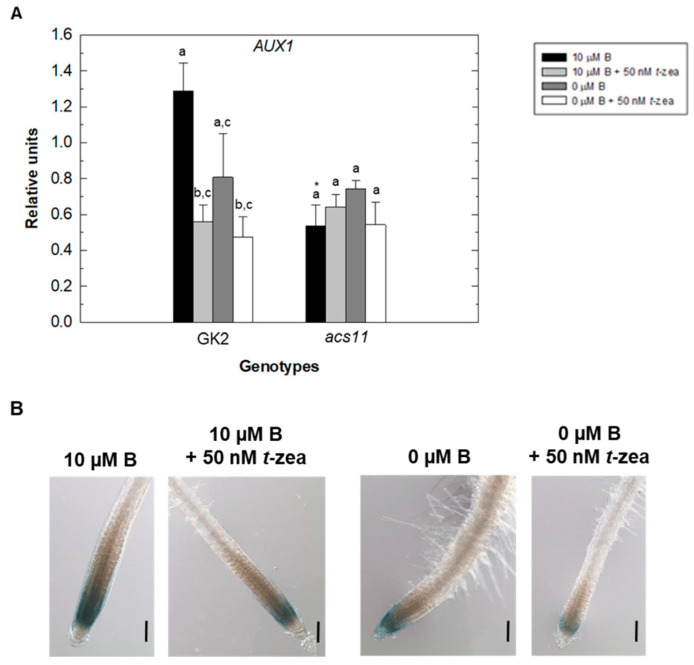
Effect of cytokinin treatment on the expression of the *AUX1* gene in Arabidopsis wild type (GK2) and *acs11* roots. (**A**) Quantitative RT-PCR analysis of *AUX1* transcript levels in control (black bars), control plus 50 nM *trans*-zeatin (light grey bars), B-deficient (dark grey bars), and B-deficient plus 50 nM *trans*-zeatin (white bars) treatment after 48 h. Results are given as means ± SD (*n* = 3 separate pools). Different letters indicate statistically significant differences between treatments for each genotype according to ANOVA with Tukey’s HSD test (*p* < 0.05). Asterisks indicate statistically significant differences between genotypes for each treatment according to Student’s *t*-test (*p* < 0.05). (**B**) GUS expression in the roots of AUX1::GUS seedlings grown in control (10 μM B) or B-deficient (0 μM B) medium in the presence or absence of *trans*-zeatin 50 nM for 48 h. Images are representative individuals from two independent experiments with at least 10 seedlings examined for each experiment. Scale bar = 100 μm.

To further explore the cross-talk between the three hormones in the control of primary root elongation under B-starvation treatment, it was tested whether *ACS11* and *AUX1* gene expression was different in the *aux1* and *acs11* mutant, respectively, when both mutants were grown in control and B-deficient treatments, in the absence and presence of *trans*-zeatin, and compared to their respective WT. For this purpose, gene expression levels in root were analyzed by quantitative RT-PCR after 48 h of treatments. The effect of *trans*-zeatin on *ACS11* and *AUX1* gene expression in both B treatments was also determined histochemically using Theo-At-ACS11-GUS/GFP and AUX1::GUS lines (Figure 6 and Figure 7).

As expected, B deficiency increased *ACS11* gene expression in the roots of WT plants after 48 h of treatment (Figure 6A). This increase was greater when *trans*-zeatin was added to the control treatment, while when added to the B-deficient treatment an increase in gene expression was also observed, although it was not statistically significant. In contrast to WT plants, there were no significant changes in root expression of the *ACS11* gene in the *aux1* mutant (Figure 6A). These results would suggest an enhancement in ACC and/or ethylene synthesis in Arabidopsis roots under exogenous *trans*-zeatin caused by increased *ACS11* expression, and that the change in auxin level caused by the alteration of the *AUX1* gene would be exerting some regulation on the expression of the *ACS11* gene. According to quantitative RT-PCR results, histochemical analysis showed an increased ACS11 activity in roots after 48 h of B deficiency and *trans*-zeatin addition (Figure 6B).

B deficiency caused a decrease in *AUX1* gene expression in the root of WT plants after 48 h of treatments (Figure 7A). In this genotype, the addition of *trans*-zeatin to both control and B-deficient treatments caused a decrease in *AUX1* gene expression, being significant in the control treatment. In contrast to WT plants, there were no changes in *AUX1* gene expression in the root of the *acs11* mutant (Figure 7A). It is noteworthy that in the control treatment, root expression levels of the *AUX1* gene in the *acs11* mutant were much lower than those in WT plants. These results would suggest that the alteration of ACC and/or ethylene synthesis caused by the absence of the *ACS11* gene would be negatively affecting *AUX1* gene expression under normal growth conditions. In the control treatment, AUX1::GUS was expressed in a discrete subset of cells in the stele, columella, lateral root cap, and epidermis (Figure 7B; [44]). Consistent with quantitative RT-PCR analysis, decreased AUX1 activity was observed in roots after 48 h of B deficiency and *trans*-zeatin addition to B treatments, leading to AUX1::GUS expression, mainly in the columella and lateral root cap (Figure 7B).

**Figure 8:**
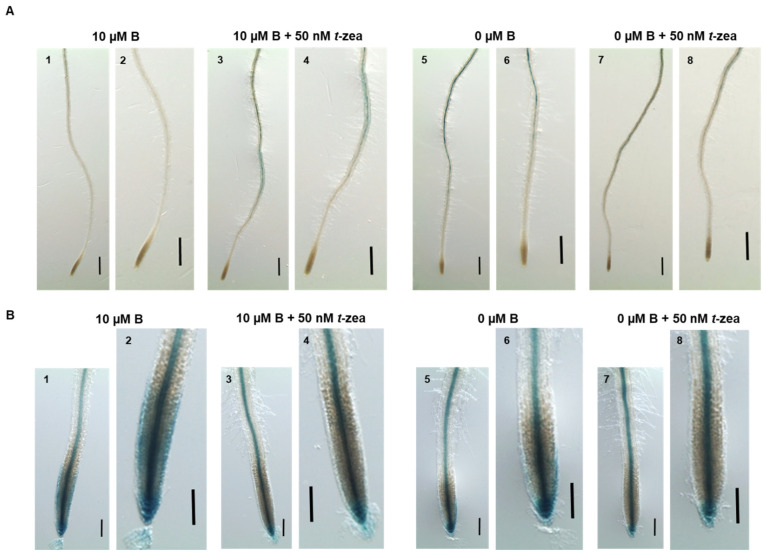
GUS expression in the roots of EBS::GUS (**A)** and IAA2::GUS (**B**) seedlings grown in control (10 μM B) or B-deficient (0 μM B) medium in the presence or absence of *trans*-zeatin 50 nM for 48 h. Images in detail are shown in the respective right sides. Images are representative individuals from two independent experiments, with at least 17 seedlings examined for each experiment. Scale bar of EBS::GUS = 500 μm, and scale bar of IAA2::GUS = 100 μm.

As the addition of *trans*-zeatin to the control and B starvation treatments led to changes in the expression of *ACS11* and *AUX1* genes, the activity of the ethylene reporter EBS::GUS and the auxin reporter IAA2::GUS was analyzed under these growth conditions (Figure 8). Upon exposure to B deficiency for 48 h, there was an increase in EBS::GUS activity in the root similar to that observed when *trans*-zeatin was added to both B treatments (Figure 8A: 3–8) and coincident with the increase in *ACS11* expression (Figure 6). In turn, B-starvation treatment partially decreased IAA2::GUS expression in the epidermis of the meristematic and, especially, elongation zones and significantly increased it in the stele and columella cells (Figure 8B: 5, 6), indicating, respectively, a decrease in auxin accumulation in this zone and an increase in rootward auxin transport from the shoot after 48 h of B deficiency. The addition of *trans*-zeatin to both B treatments completely decreased the IAA2::GUS signal in the epidermis of the meristematic and elongation zones and caused an increase in the signal in the stele like that observed in response to B deficiency (Figure 8B: 3, 4; 7, 8). These results are consistent with those obtained for the expression levels of *AUX1* (Figure 7).

## 3. Discussion

There are studies supporting the concept that cytokinin regulates root growth inhibition under B deficiency by controlling meristem cell proliferation in Arabidopsis plants [34,35]. In addition, it has also been described that an interaction between ethylene and auxin plays an important role in controlling the Arabidopsis primary root elongation under short-term B deprivation [31,32,33]. Our results suggest that cytokinin would be regulating primary root elongation under B-starvation conditions, because an increased GUS signal was observed in the ARR5::GUS reporter line (Figure 1A) and, in addition, there was a lower inhibition in primary root elongation in the *ahk4* mutant (Figure 1B), which is affected in cytokinin perception. Therefore, the possible interaction among cytokinin, ethylene, and auxin in the response of the primary root to B-deprivation treatment is studied herein. It is noteworthy that this same ARR5::GUS reporter line has been previously used to record endogenous cytokinin status [45,46,47,48]. ARR5 is a type-A ARRs that is transcriptionally induced in response to cytokinin [49]. Our results suggest that in B deficiency the increased GUS signal in the ARR5::GUS reporter line in the stele and meristematic region of the root (Figure 1A) would be associated with an increase in endogenous cytokinin in this area that would be causing an inhibition of primary root elongation less evident in the *ahk4* mutant (Figure 1B).

It is well known that, under normal growth conditions, cytokinins negatively regulate root growth by both reducing meristem size owing to control of cell differentiation and inhibiting cell elongation [50,51]. In the Arabidopsis root, the ability of cytokinins to inhibit cell elongation, but not cell proliferation in the radical meristem, depends on the auxin importer AUX1. In fact, this carrier is required for cytokinin-dependent auxin activity changes in the lateral root cap associated with the control of cell elongation [40]. Our results showed that, in comparison to the control treatment, the strong inhibition of LEH in WT plants under B deficiency and when *trans*-zeatin was added to both B treatments did not appear in the *aux1* mutant. This LEH inhibition led to a reduction of primary root elongation in WT plants (Figure 2). However, in the mutant, primary root elongation was much more negatively affected than LEH by the B-deficient treatments (Figure 2A), suggesting that this reduction in primary root growth is mainly due to altered cell proliferation caused by changes in cytokinin signaling in the meristematic region under these conditions ([35]; Figure 1A). It should be noted that the increase in both primary root elongation (Figure 2A) and LEH in WT plants (Figure 2B) after 48 h of adding *trans*-zeatin to the B-deficient treatment (Figure 2A) could be attributed to the cytokinin resistance conferred by the overexpression of type-A ARRs, such as ARR5, described by To et al. [52]. These authors overexpressed different type-A ARRs (including ARR5) in WT Arabidopsis plants and found that all transgenic lines tested were significantly more resistant to different cytokinin concentrations than the WT in root elongation assays [52]. Therefore, the increased GUS signal in the roots of the ARR5::GUS reporter line under B deficiency (Figure 1A) would confer resistance when *trans*-zeatin was added under these conditions, resulting in increased primary root elongation and LEH (Figure 2). The correlation between the results of LEH and primary root elongation suggests that the increase in endogenous cytokinin in the stele and meristematic region of the root reported by ARR5::GUS in B starvation treatment (Figure 1A) would be causing an inhibition of root cell elongation that would lead to reduced root growth (Figure 2). This decrease of root growth would be mediated through the auxin influx-carrier AUX1, as suggested by the lower inhibition of root elongation observed in the *aux1* mutant under B deficiency (Figure 2A). In addition, the cytokinin accumulation under B deficiency would be negatively affecting *AUX1* expression, as did the addition of *trans*-zeatin to the growth medium (Figure 7), previously described by Street et al. [40], which caused an alteration of auxin activity in the stele and meristematic and elongation zones of the root (Figure 8B), affecting cell elongation. Camacho-Cristóbal et al. [32] have already reported repression of root *AUX1* expression under short-term B deficiency (4 h).

The inhibitory effects of cytokinin on root cell elongation also depend on ethylene [40]. The role of ethylene in cytokinin regulation of root cell elongation has been proposed on the basis of two facts: (1) the ability of cytokinin to induce ethylene biosynthesis by increasing the stability of ACS5 and ACS9 isoforms, both type 2 ACSs [36,38], and (2) the finding that ethylene inhibits root growth through effects on cell elongation [41,53] and that it requires, like cytokinin, AUX1-dependent changes in auxin distribution [54,55]. In turn, ethylene increases the expression of auxin influx-carrier AUX1, enhancing the basipetal auxin transport and stimulates auxin biosynthesis, which is transported toward the root tip (see Figure 8 in [54]), facts that support the significant reduction in *AUX1* gene expression observed in the *acs11* mutant under control conditions (Figure 7A). In addition, this reduction would lead to altered auxin response in the primary root of the *acs11* mutant, which, in turn, would result in decreased root cell length (Figure 3; [55,56]). Our results showed that adding *trans*-zeatin to both B treatments increased the *ACS11* gene expression in the root, especially in the control treatment (Figure 6). This gene was also induced by B deficiency (Figure 6; [32]), perhaps due in part to the accumulation of cytokinin that occurred in this B treatment. The results in the *aux1* mutant showed that an alteration of auxin content also affected the level of *ACS11* transcripts (Figure 6A). Thus, it has been described that *ACS11* gene expression is strongly induced in the root after the addition of IAA [57]. Interestingly, ACS11 is a type 2 ACS [58], like ACS5 and ACS9, and an increase in its expression levels caused an enhancement in ethylene biosynthesis, as observed in Figure 8A. This figure shows increased GUS activity in the ethylene reporter line EBS::GUS under B-deficient treatment (Figure 8A: 5, 6) and after the addition of *trans*-zeatin to the different B media (Figure 8A: 3, 4; 7, 8). However, this increase in ethylene content in the Arabidopsis root, in contrast to that described by Růžička et al. [54], did not favor the expression of *AUX1*, which is decreased by the presence of cytokinin, as discussed above (Figure 7). However, the rise in ethylene could contribute to the increased auxin levels observed in the stele, as shown by the activity results of the auxin reporter IAA2::GUS (Figure 8B). The lower effect on root growth caused by B deficiency in the *acs11* mutant could be because cytokinin only acts through ethylene-independent mechanisms (Figure 3; [40]).

Taking into account the fact that B deficiency causes a decrease in primary root cell elongation mediated by cytokinin, ethylene, and auxin, and that the best-known role of B is its structural function in the cell wall [16,17,18], it is reasonable to think that these hormones may be among the factors involved in B uptake and transport. In this regard, a regulatory role of phytohormones in the *NIP5;1* promoter of Arabidopsis plants has recently been shown, which may affect B transport [59]. The higher B content in both roots and shoots of the *aux1* mutant compared to WT in the control treatment would not be explained by changes in the B transporters analyzed (Figure 4), so that in this mutant B would mainly enter the root through other B-channels/transporters [60,61] different from NIP5;1, which introduces B into root cells and, in addition, is required for efficient uptake of this nutrient under B limitation [62]. The higher B content in the roots of the mutant under B-deficient conditions despite lower levels of *NIP5;1* transcripts supports this idea (Figure 4A,E). The lower shoot B content in the *aux1* mutant compared to WT under B starvation could be explained by the higher *BOR2* gene expression in the roots of this mutant. It has been proposed that boric acid/borate transport by BOR2 from the symplast to the apoplast in roots is required for effective cross-linking of RG-II in the cell wall and root cell elongation [63], which would limit the B delivered in shoots. Interestingly, under low B conditions, both NIP5;1 and BOR2 are expressed in the lateral root cap ─among other locations─ [63,64,65], as well as AUX1 [44]. Therefore, the alteration of auxin signaling in this area in the *aux1* mutant would be favoring the expression of *BOR2* and reducing that of *NIP5;1*, suggesting a possible role of auxin in the regulation of the expression of these genes. Gómez-Soto et al. [59] already suggested that auxins might be affecting the expression of the *NIP5;1* promoter in the elongation zone under B deficiency. Furthermore, it has also been described that the addition of the ethylene precursor 1-aminocyclopropane-1-carboxylic acid (ACC) caused an induction of Arabidopsis *NIP5;1* gene expression, although it did not correlate with higher B concentrations [59], suggesting that increased ethylene in roots positively affects the expression of this B transporter. In the *acs11* mutant, B content in roots and shoots was higher than in WT in the control treatment, while the expression of all B transporters analyzed decreased, especially *BOR1* and *NIP5;1* (Figure 5). BOR1 encodes an efflux-type B transporter that is required for effective xylem loading under B-limited conditions [66], and NIP5;1 is needed for efficient uptake of this micronutrient also under B starvation [62]. Our results support the importance of ethylene in the regulation of *NIP5;1* expression (Figure 5E; [59]), and also suggest that this hormone could be involved in the regulation of the expression of other B transporters under control conditions (Figure 5). In control B treatment, the lower expression levels of B transporter genes in the *acs11* mutant and its higher root B level compared to those of WT suggest that the B uptake in this mutant is mainly occurring by passive transport that would be reflected in the increased B content in shoots (Figure 5). Despite the absence of B in the growth medium, WT accumulated this nutrient in roots, probably due to a reduction in xylem loading by *BOR1*, whose expression was repressed under these conditions (Figure 5A,C). This fact would corroborate the low B content found in the shoots under B deficiency. Surprisingly, *acs11* had a high B content in the shoots when this micronutrient was absent from the culture medium, despite its lower root B content.

Additionally, as discussed above, the absence of the auxin influx-carrier AUX1 in the *aux1* mutant negatively affected *NIP5;1* transcript levels under B starvation when compared with the WT. This fact is consistent with the low levels of *AUX1* gene expression observed in the *acs11* mutant under control conditions where *NIP5;1* gene expression was also negatively affected (Figure 4E, Figure 5E and Figure 7A). Therefore, the balance between ethylene and auxin in roots could be involved in the regulation of *NIP5;1* gene expression, both in the absence and presence of adequate B conditions. Cytokinins are known to influence the uptake of macronutrients, such as nitrogen, phosphorus, sulfur, and potassium. For example, exogenous application of cytokinin represses several genes of nitrate transporter *AtNRT* and three genes of the ammonium transporter *AtAMT* that regulate nitrogen uptake [67]. For phosphorus and sulfur, cytokinins can negatively regulate phosphate and sulfate uptake by controlling the expression of the *AtPT1* gene encoding a phosphate transporter or the *SULTR1;1* and *SULTR1;2* genes encoding sulfate transporters [23,29]. Cytokinins also negatively regulate gene expression of the high-affinity K transporter *HAK5* in response to potassium starvation [30]. Our results showed a negative regulatory role of cytokinins on the gene expression of *NIP6;1* and *BOR1* transporters in both WT and *aux1* mutants, which would indicate that this effect of cytokinins would be independent of altered auxin signaling (Figure 4). Similar results were obtained when GK2 was used as the wild-type genotype (Figure 5). The differences found in the expression of B transporters in Col 0 and GK2 strains would be due to minor genetic alterations in the two Arabidopsis lines.

## 4. Materials and Methods

### 4.1. Plant Materials and Growth Conditions

*Arabidopsis thaliana* ecotype Columbia (Col 0), *aux1* mutant (N9585), *ahk4* mutant (N661880), and Theo-At-ACS11-GUS/GFP transgenic line (N31387) were obtained from the Nottingham *Arabidopsis* Stock Centre (NASC) (https://arabidopsis.info/BasicForm). *Arabidopsis thaliana* ecotype GK2 (wild type) and the *acs11* mutant (GABI_284B12) were kindly provided by Dr J. Keurentjes (Laboratory of Genetics, Wageningen University, Wageningen, The Netherlands). The following GUS reporter lines were also used in this study: AUX1::GUS (provided by Dr R. Swarup, School of Biosciences and Centre for Plant Integrative Biology, University of Nottingham, Nottingham, UK), ARR5::GUS and IAA2::GUS (provided by Dr P. Doumas, INRA, Montpellier, France), and EBS::GUS (provided by Dr J. Alonso, Department of Genetics, North Carolina State University, Raleigh, NC, USA).

Seeds were surface-sterilized with 75% (*v*/*v*) ethanol for 5 min, then with 2% (*w*/*v*) hypochlorite solution for 5 min and, finally, washed six times with sterile water. Sterile seeds were sown on square (12 × 12 cm) Petri dishes containing 40 mL of sterile culture medium and sealed with Parafilm. The culture medium contained 1 mM Ca(NO_3_)_2_, 1 mM KNO_3_, 0.5 mM MgSO_4_, 0.75 mM KH_2_PO_4_, 12.5 μM NaCl, 12.5 μM FeNa-EDTA, 2.5 μM MnCl_2_, 0.5 μM ZnSO_4_, 0.25 μM CuSO_4_, 0.125 μM Na_2_MoO_4_, 0.05 μM CoCl_2_, 10 μM H_3_BO_3_, 2 mM MES, and 0.5 % (*w*/*v*) sucrose, adjusted to pH 5.7 with KOH and solidified with 1% (*w*/*v*) Phytagel. After incubation at 4 °C for 5 days in the dark to promote and synchronize seed germination, the dishes were placed vertically in a growth chamber under a light/dark regime of 16/8 h, 23/22 °C, 70/70% relative humidity, and a light intensity of 120–150 μmol·m^−2^·s^−1^ photosynthetically active radiation.

Seedlings were grown under these conditions for 5 days and then carefully transferred to control medium (10 μM B) or B-deficient medium (no B addition) for 48 h. When indicated, 50 nM *trans*-zeatin was added to the medium before solidification.

Analytical-grade compounds were always used to prepare the nutrient solutions and reagents. Purified water was obtained by a system consisting of three units (active charcoal, ion exchanger, and reverse osmosis) connected in series to an ELGA water purification system (PURELAB ultra), which supplied water with an electrical resistivity of 18.2 MΩ cm.

### 4.2. Root Length, Elongation and LEH Measurements

Images of the root system were recorded directly from plants growing in Petri dishes using a desktop scanner (resolution: 200 dpi). Images corresponding to different growth times were analyzed with Optimas version 6.1 software (Media Cybernetics, Rockville, MD, USA). The length of the primary root was determined manually. Data were exported to an Excel worksheet for final processing. Primary root elongation was calculated by subtracting the primary root length at time 0 from the primary root length at the indicated time.

After 48 h of treatments, the measurement of the length of the first epidermal cell with a visible root hair bulge (LEH; [41,42]) was determined, as described by Camacho-Cristóbal et al. [32].

### 4.3. Boron Determination

Frozen root or shoot pools were ground to a fine powder with liquid nitrogen, and aliquots of 100 mg were burnt to ashes at 550 °C in a muffle furnace for 6 h. Ashes, once at room temperature within a desiccator, were dissolved with 0.1 M HCl, and total B concentrations were determined by the azomethine-H method, as described by Beato et al. [68].

### 4.4. RNA Isolation, cDNA Synthesis, and Quantitative RT-PCR Analyses

Total RNA was extracted using Tri-Reagent RNA/DNA/Protein Isolation Reagent (Molecular Research Center, Cincinnati, OH, USA) and then treated with RNase-free DNase (Qiagen, Hilden, Germany) according to the manufacturer’s instructions. RNA was purified using an RNA Clean & Concentrator column (Zymo Research, Irvine, CA, USA). Two micrograms of DNase-treated total RNA was used to prepare cDNA by reverse transcription with M-MLV reverse transcriptase (New England Biolabs Inc., Ipswich, MA, USA) and oligo(dT)18 primers (Bioline, London, UK), according to the manufacturer’s protocol. Gene expression was determined by quantitative RT-PCR (MyiQ real-time PCR detection system, Bio-Rad Laboratories Inc., Hercules, CA, USA) using gene-specific primers (Table S1) and SensiMix SYBR & Fluorescein Kit (Bioline, London, UK), following the manufacturer’s instructions.

The Arabidopsis *TON1A* amplicon (At3g55000) was used as an internal control to normalize all data. The efficiency of the quantitative RT-PCR reactions was higher than 95%.

Quantitative RT-PCR reactions were carried out with cDNA synthesized from three pools of 36–60 roots, depending on the treatment used, harvested randomly.

### 4.5. Histochemical Analysis

For histochemical analysis of β-glucuronidase (GUS) reporter enzyme activity, seedlings of the reporter lines were incubated at 37 °C in GUS reaction buffer containing 2 mM 5-bromo-4-chloro-3-indolylb-D-glucuronide in 100 mM sodium phosphate (pH 7.0). GUS staining patterns were analyzed on a Leica S8APO Stereozoom microscope equipped with a digital camera (Leica EC3) and driven by analysis software (LAS EZ; Leica, Wetzlar, Germany).

For each marker line and for each treatment, at least nine transgenic plants from two independent experiments were analyzed. Representative plant images were chosen for each treatment.

### 4.6. Statistical Analysis

Data shown are mean values ± SD. All experiments were repeated at least twice. Results were statistically analyzed using one-way analysis of variance (ANOVA). Differences among treatment means were evaluated by Tukey’s Honestly Significant Difference test. Student’s *t*-test was used for comparison between two means.

## 5. Conclusions

**Figure 9:**
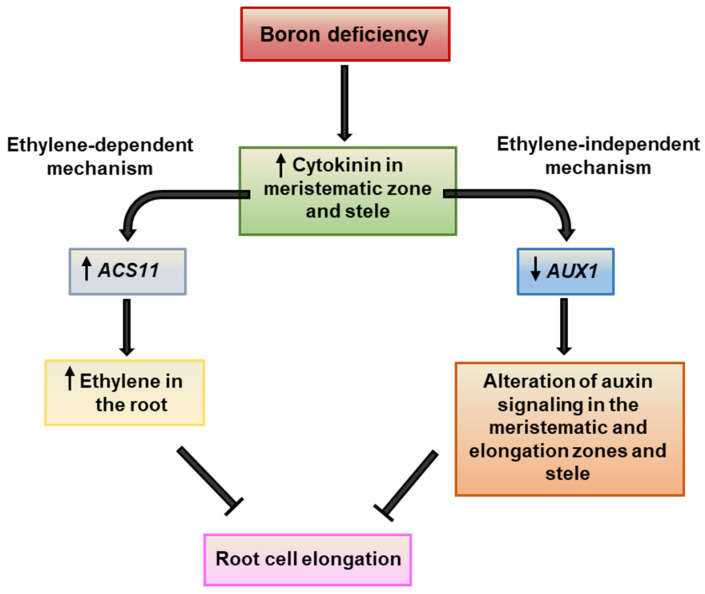
Model for cytokinin inhibition of root cell elongation under B deficiency in *Arabidopsis thaliana* plants. For more details, see the text.

In conclusion, this study provides evidence that cytokinins negatively regulate root cell elongation in Arabidopsis plants subjected to B deficiency through two pathways: (i) an increase in ethylene synthesis caused by the induction of the *ACS11* gene, and (ii) an alteration of auxin activity in the meristematic and elongation zones and stele, by repression of *AUX1* gene expression (Figure 9). Moreover, under these B starvation conditions, altered auxin signaling would positively affect *BOR2* expression and reduce *NIP5;1* expression, whereas under adequate B conditions, ethylene would be the main hormone in the regulation of B transporters, especially *NIP5;1* and *BOR1*. Therefore, the balance between ethylene and auxin in the root could be involved in the regulation of *NIP5;1* expression, in both the absence and the presence of adequate B supply. Finally, cytokinins would have a negative regulatory role on the expression of *NIP6;1* and *BOR1* transporters that would be independent of altered auxin signaling. Further studies should be performed to validate the exact role of these hormones in the regulation of B transport.

## Data Availability

The data presented in this study are available in the text and supplemental data.

## Supplementary Materials

The following supporting information can be downloaded at: https://www.mdpi.com/article/10.3390/plants11182344/s1, Figure S1: Dose-response curve of primary root elongation to external concentrations of *trans*-zeatin in control (black bars) and B-deficient (white bars) after 24 h (**A**), 48 h (**B**), 72 h (**C**), and 96 h (**D**) treatments with B; Figure S2: Effect of cytokinin treatment on primary root length of Arabidopsis wild type (Col 0) and *aux1* mutant under B deficiency; Figure S3: Effect of cytokinin treatment on primary root length of Arabidopsis wild type (GK2) and *acs11* mutant under B deficiency; Table S1: List of primers used in this study for quantitative RT-PCR analyses.
