# Supplementary material for: Crosstalk of Cytokinin with Ethylene and Auxin for Cell Elongation Inhibition and Boron Transport in Arabidopsis Primary Root under Boron Deficiency
Source: Plants (Basel). 2022 Sep 8;11(18):2344. doi: 10.3390/plants11182344 (PMC9504276; doi:10.3390/plants11182344)
Supplement: Supplementary file 1 [file plants-11-02344-s001.zip › plants-1861706-supplementary/Figure S1.pdf]

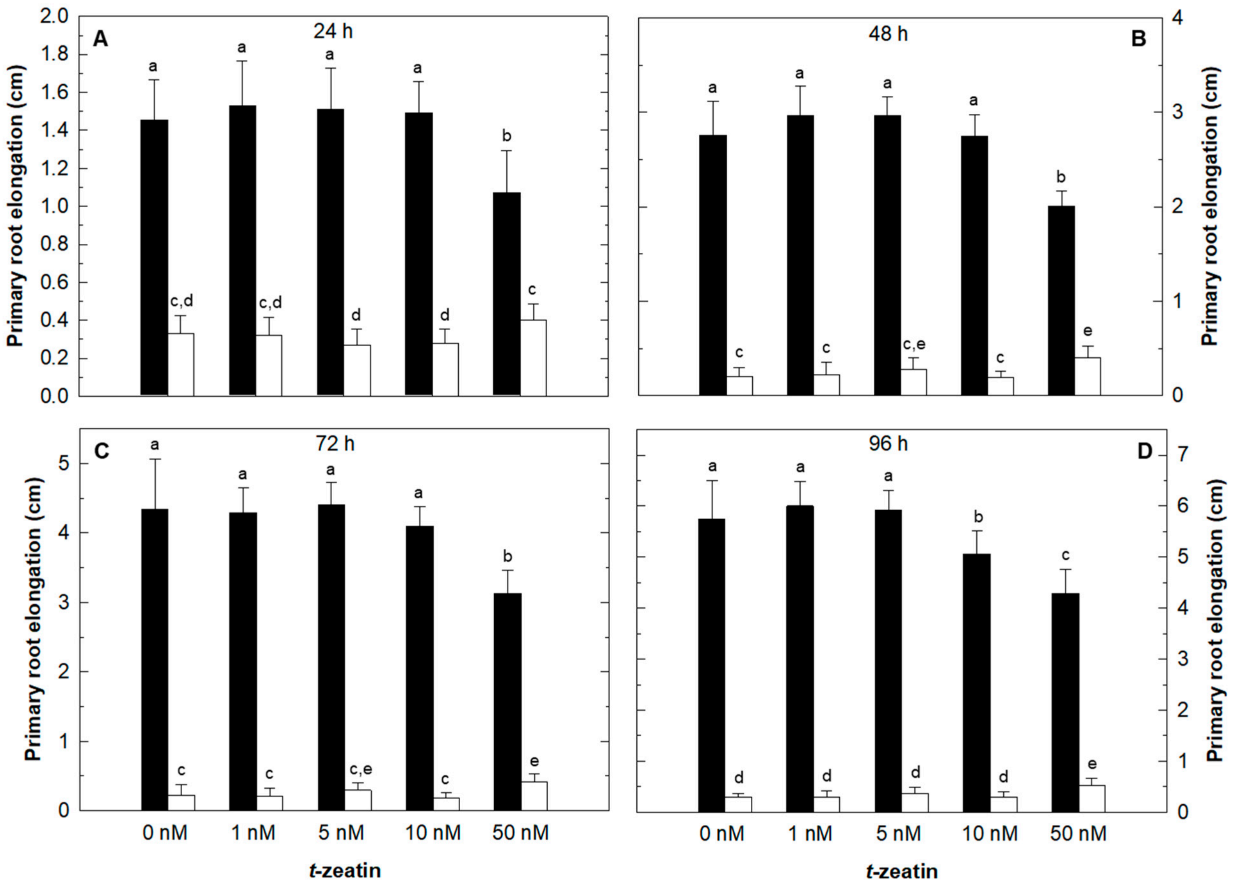

Figure S1. Dose-response curve of primary root elongation to external concentrations of *trans*-zeatin in control (black bars) and B-deficient (white bars) after 24 h **(A)**, 48 h **(B)**, 72 h **(C)** and 96 h **(D)** treatments with B. Results are given as means ± SD (n = 24 separate plants). Different letters indicate statistically significant differences between treatments according to ANOVA with Tukey's HSD test ($P < 0.01$).
