# Supplementary material for: Crosstalk of Cytokinin with Ethylene and Auxin for Cell Elongation Inhibition and Boron Transport in Arabidopsis Primary Root under Boron Deficiency
Source: Plants (Basel). 2022 Sep 8;11(18):2344. doi: 10.3390/plants11182344 (PMC9504276; doi:10.3390/plants11182344)
Supplement: Supplementary file 1 [file plants-11-02344-s001.zip › plants-1861706-supplementary/Figure S3.pdf]

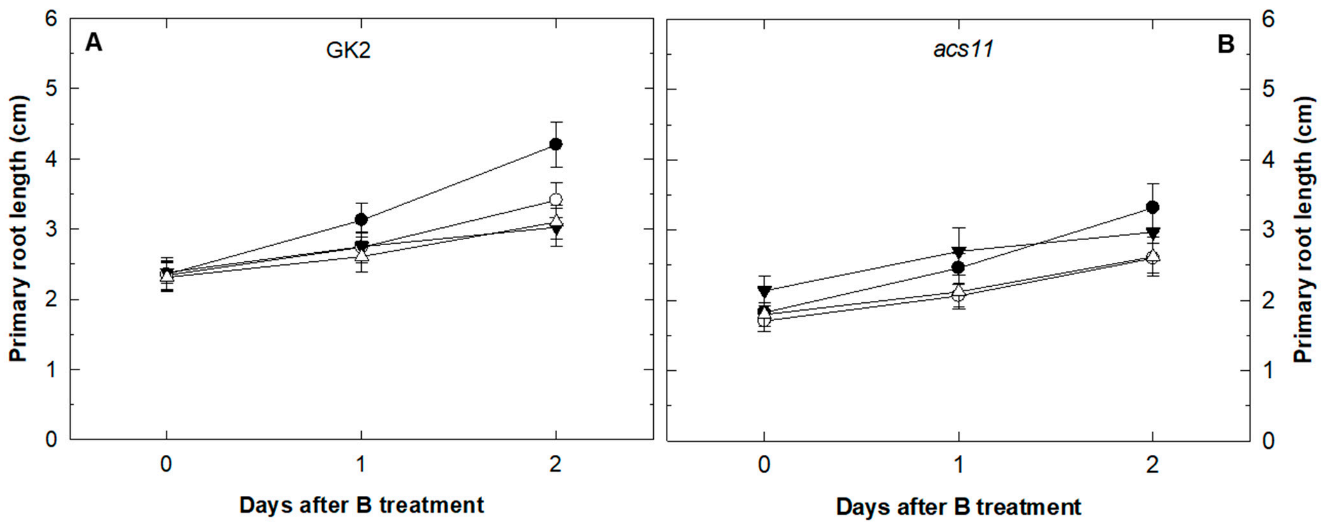

Figure S3. Effect of cytokinin treatment on primary root length of Arabidopsis wild type (GK2) and *acs11* mutant under B deficiency. Time-course of primary root length was measured in Arabidopsis wild type (GK2) **(A)** and *acs11* mutant **(B)** in control (filled circles), control plus 50 nM *trans*-zeatin (open circles), B-deficient (filled triangles), and B-deficient plus 50 nM *trans*-zeatin (open triangles) treatment for 2 days. Results are given as means ±SD (n=24 separate plants).
