# Supplementary material for: Crosstalk of Cytokinin with Ethylene and Auxin for Cell Elongation Inhibition and Boron Transport in Arabidopsis Primary Root under Boron Deficiency
Source: Plants (Basel). 2022 Sep 8;11(18):2344. doi: 10.3390/plants11182344 (PMC9504276; doi:10.3390/plants11182344)
Supplement: Supplementary file 1 [file plants-11-02344-s001.zip › plants-1861706-supplementary/Supplementary Table S1.pdf]

Supplementary Table S1. List of primers used in this study for quantitative RT-PCR analyses.

Gene	Forward primer	Reverse primer
TON1A	5′-TGTGAGGGATGGAACAAATG-3′	5′-AACGCAGTTGCAAATAAAGGA-3′
ACS11	5′- ACAGCTGGATCAACCTCGGCT-3′	5′- GTCGCGGCTGACACCACTTTCT-3′
AUX1	5′- AGACGCACTTCTCGACCACTCCA-3′	5′- GCATCCCAATCACTTTCTCCCACA-3′
BOR1	5′- AATCTCGCAGCGGAAACG-3′	5′- TGGAGTCGAACTTGAACTTGTC-3′
BOR2	5′- TTCAGGACTTAGAGCCAGCA-3′	5′- ACGAAGCAGTTGATCCCATC-3′
NIP5;1	5′- CACCGATTTTCCCTCTCCTGAT-3′	5′- GCATGCAGCGTTACCGATTA-3′
NIP6;1	5′- GGCAATGGTTACAGCCGGAT-3′	5′- GGAGCTGAGACGCTTATTGGTT-3′
